# From Stem Cells to Populations—Using hiPSC, Next-Generation Sequencing, and GWAS to Explore the Genetic and Molecular Mechanisms of Congenital Heart Defects

**DOI:** 10.3390/genes12060921

**Published:** 2021-06-16

**Authors:** Martin Broberg, Johanna Hästbacka, Emmi Helle

**Affiliations:** 1 Stem Cells and Metabolism Research Program, Faculty of Medicine, University of Helsinki, 00290 Helsinki, Finland; martin.broberg@helsinki.fi; 2 Institute for Molecular Medicine Finland, HiLIFE, University of Helsinki, 00290 Helsinki, Finland; 3 New Children’s Hospital, and Pediatric Research Center, Department of Anesthesia and Intensive Care, Helsinki University Hospital, 00290 Helsinki, Finland; johanna.r.hastbacka@hus.fi; 4 New Children’s Hospital, and Pediatric Research Center, Department of Pediatrics, Helsinki University Hospital, 00290 Helsinki, Finland

**Keywords:** congenital heart disease, genetics, genome-wide association studies, massively parallel sequencing, human induced pluripotent stem cells

## Abstract

Congenital heart defects (CHD) are developmental malformations affecting the heart and the great vessels. Early heart development requires temporally regulated crosstalk between multiple cell types, signaling pathways, and mechanical forces of early blood flow. While both genetic and environmental factors have been recognized to be involved, identifying causal genes in non-syndromic CHD has been difficult. While variants following Mendelian inheritance have been identified by linkage analysis in a few families with multiple affected members, the inheritance pattern in most familial cases is complex, with reduced penetrance and variable expressivity. Furthermore, most non-syndromic CHD are sporadic. Improved sequencing technologies and large biobank collections have enabled genome-wide association studies (GWAS) in non-syndromic CHD. The ability to generate human to create human induced pluripotent stem cells (hiPSC) and further differentiate them to organotypic cells enables further exploration of genotype–phenotype correlations in patient-derived cells. Here we review how these technologies can be used in unraveling the genetics and molecular mechanisms of heart development.

## 1. Introduction

Congenital heart defects (CHD) are the most common form of congenital malformations, affecting 0.8–1% of the population [1]. CHD may occur as an element of a syndrome or as an isolated condition. Non-syndromic CHD (nsCHD) can be familial or sporadic. In familial forms, nsCHD is characterized by incomplete penetrance and variable expressivity [2,3], and an inherited etiology can be identified in approximately one-third to half of the familial cases [4,5,6]. By contrast, in most sporadic nsCHD cases, a genetic etiology has not been identified with only ca. 10% representing de novo genetic variants and 3–10% copy number variation [7,8]. It appears that nsCHD is often oligogenic, with two or more predisposing genetic variants contributing to disease [9,10]. An interesting recent study using Mendelian Randomization (MR) proposed a new heritable form of risk for CHD originating from inherited extremes in the size of developing cardiovascular anatomy, where inheritance of a smaller diameter of the ascending aorta corresponded to an increase in risk for left ventricular outflow tract (LVOT) CHD [11]. In addition to genetic variations, nsCHD risk factors include epigenetic changes [12] and adverse environmental stimuli, such as maternal glycemic dysregulation, obesity, and certain medications and infections during pregnancy [13,14,15,16].

New technologies have paved the way to an improved understanding of CHD. Next-generation sequencing and genome-wide association studies (GWAS) have expanded our understanding of the etiological factors of CHD. Patient-derived human-induced pluripotent stem cells (hiPSCs) provide an excellent tool for disease modeling. Genetic factors have also been identified to impact disease course [17,18,19], expanding the clinical significance of understanding their impact on CHD. In this review, we discuss how these methods can be used to identify new genes and signaling pathways underlying CHD and to explore how genetic variants impact disease prognosis and outcome of care.

## 2. Identification of Causative Genes by Linkage Analysis and Chromosomal Microarray

The first genes associated with CHD were identified in 1998 by linkage analysis in families with multiple affected members. Pathogenic dominant *NKX2-5* variants were identified in four families with a history of secundum atrioventricular septal defects (ASD), ventricular septal defects (VSD), subvalvular aortic stenosis (SVAS), Tetralogy of Fallot (TOF) with pulmonary atresia (PA), and atrioventricular conduction abnormalities [3]. *GATA4* was identified as a causal dominant variant in a family with recurring septal defects and insufficiency of cardiac valves [20]. *NOTCH1* was identified by linkage analysis to cause left ventricular outflow tract obstruction (LVOTO) defects and other CHD [21]. In addition to nsCHD, *TBX5* and *TBX20* were shown by this technique to cause CHD with malformations of other organ systems [22,23].

During the early 2000s, the development of array comparative genomic hybridization enabled high-resolution genome-wide screening for copy number variants (CNVs) [24]. Pathogenic CNVs have been identified in ca. 10% of nsCHDs, and an excess burden rate of rare CNVs in nsCHD patients compared with healthy controls has been noted in several studies [19,25,26,27]. The observation that CNVs are predominantly associated with CHD with extracardiac anomalies [28,29] may be due to the altered function of multiple genes by the CNV. Besides influencing the transcription of the genes localized within the CNV, CNVs can regulate the expression of target genes at a distance [30]. Chromosomal microarray is usually the first-line genetic test done in the clinical setting to identify CNVs in CHD with extracardiac anomalies and in individuals with conotruncal defects or type A interruption of the aortic arch as these can be the only early manifestation of 22q11-deletion syndrome.

## 3. Next-Generation Sequencing as a Tool for CHD

The majority of known large effect variants contributing to genetic conditions reside in coding DNA. Therefore, selectively sequencing the exons of protein-coding genes, whole-exome sequencing (WES), has become a prudent approach to variant detection in conditions with suspected genetic etiology. In addition to research applications, WES is widely used in clinical diagnostics.

WES can be applied both on familial and sporadic forms of disease. It is plausible that a considerable share of sporadic CHD represents de novo variants. This is because severe CHD reduces reproductive fitness and has a relatively low sibling recurrence risk. And yet, the incidence of CHD remains constant. WES sequencing of proband-parent trios is an effective approach to identify de novo variants. Indeed, in an early study, a marked increase in de novo coding variants in genes highly expressed in a developing heart was noted in 362 child–parent trios with severe CHD compared to 264 control trios [7]. By filtering out missense mutations, the variants associated with disease increased from odds ratio (OR) 2.53 to 7.50. More than half of the patients in this study had extracardiac congenital anomalies, developmental, or growth issues. The de novo variant positivity was less frequent in patients with isolated CHD, which is in accordance with several later WES studies showing that damaging de novo variants are significantly more prevalent in patients with other anomalies and/or neurodevelopmental defects, whereas there is an excess of inherited damaging mutations in patients with isolated CHD [18,31,32]. Moreover, an excess of predicted deleterious missense and loss of function de novo variants have been observed in patients with conotruncal defects, left ventricular outflow tract defects, and in patients classified to belong to the “other” category of CHD, but not in patients with heterotaxy [32] While the genetic basis of CHD is still mostly unsolved, the familial contribution has been shown to vary between different CHD types and is highest in heterotaxy, in which, on the other hand, de novo variants are uncommon [7,32,33,34].

As an aid to interpreting variants in the large WES datasets, Szot et al. [6] curated a list of 90 high-confidence CHD genes known to cause human CHD when analyzing the exomes of 30 CHD families and identified a pathogenic variant in 10% of families and a likely disease-causing variant in 33% of the rest [6]. The list maintained by Victor Chang Cardiac Research Institute (http://chdgene.victorchang.edu.au, accessed on 5 May 2021) currently includes 133 genes. In a WES study of patients with left-sided lesions, Li et al. [35] generated a list of 1760 candidate genes from the literature and model organism databases identifying a candidate variant in 17 novel and 10 known CHD genes in 14.3% of cases [35].

The yield of variant detection varies widely and depends on the selection criteria of CHD patients and the stringency of interpretation. Detection of CHD causing variants has ranged from 2 to 46% [4,5,31,32,36] and is higher in CHD with extracardiac features than in isolated CHD. It is apparent that a portion of disease-causing variants lie beyond the exome, suggesting that the increasingly applied whole-genome sequencing (WGS) could provide additional information. In addition to full coverage of the genome, including its regulatory sequence, the “regulome”, WGS enables more comprehensive detection of most types of genomic variation than WES (single nucleotide changes, small insertions and deletions, and CNVs and some of the structural variation). The pathogenicity of variants in a non-coding sequence is, however, significantly more difficult to establish than that of variants in a coding sequence.

The new information WGS has provided to CHD is variable. Reuter et al. analyzed 111 trios or families with a CHD child [37], excluding patients with a designated syndrome or metabolic disorder. In the cases where still nearly half of the patients had extracardiac features, a causative variant was identified in 14 families. Eleven patients with diagnostic variants had extracardiac features, and two had a family history of CHD. By mitigating the variant interpretation criteria, which were based on American College of Medical Genetics and Genomics (ACMG) guidelines and evaluation by a clinical geneticist, a genetic counselor and a cardiologist, to include variants that appeared plausible but did not meet the criteria for pathogenicity, the number of potentially relevant variants increased from 14 to 22 without decreasing the proportion of CHD with extracardiac issues. Seven of the variants were CNVs, two of which included genes with a known contribution to CHD. Variants involved 17 different genes, two of which were found twice in separate patients with different cardiac phenotypes. All detected single nucleotide variants (SNV) were in the coding DNA or in splice sites and therefore could have been detectable also by WES. Another WGS study of 24 critically ill newborn infants with CHD and other malformations identified a definitive or likely genetic diagnosis in 11 patients [38]. Some of the probands were also tested with chromosomal microarray and targeted gene panels recommended by their primary medical team. Not surprisingly, the diagnostic yield was higher in WGS. However, excluding a 3 Mb deletion including an established CHD gene, all variants detected were in coding DNA and could have also been detectable by less laborious WES. Low read-depth (≈0.6×) WGS has also been successfully used to search for de novo CNVs in non-syndromic atrioventricular septal defects [39].

Although it is known that in the familial forms, CHD phenotypes can differ between affected family members [40], it is still more likely that the phenotypes of affected family members are concordant [2,33]. In addition, variants of certain genes are more often associated with a certain phenotype, such as *NOTCH1* variants with LVOTO defects [2,21,41]. Thus, it is prudent to search for new candidate genes and additional data for their pathogenicity from the related gene pathways of known disease genes. This approach was taken in the genome sequencing study of 175 TOF patients in which VEGF-pathway genes were studied [42], replicating the results of an earlier study indicating that the haploinsufficiency of *FLT4* associates with TOF [34]. In addition, *KDR* loss-of-function variants and likely disturbing variants in five other VEGF-pathway genes were identified, strengthening the hypothesis of dysregulated VEGF-signaling contributing to the pathogenesis of TOF [42].

By using complex computational and statistical methods, the evaluation of variants in non-coding regions has been improved. A WGS study on 763 trios who had not shown rare damaging missense or loss-of-function coding variants in known CHD genes found evidence of potentially disruptive regulatory non-coding de novo-variation being at least as common as a damaging de novo-variation in coding DNA [43]. In this study, 40.8% of the probands had neurodevelopmental defects and/or extracardiac anomalies. Interestingly, the findings were similar in probands with isolated CHD and probands with extracardiac anomalies in contrast to the damaging coding de novo variant enrichment seen among CHD probands with extracardiac anomalies. Whether variants in non-coding regions are capable of causing CHD in a Mendelian model remains to be elucidated.

The data analysis in WGS is remarkably more demanding than in WES with a 50–100 fold amount of sequence with deficiently understood biological significance to be analyzed. Indeed, in many WGS studies, the variants considered disease-causing have resided in the coding sequence and therefore could have been detected by WES, although there is data indicating that WGS provides better exome coverage than WES [44]. The true utilization of WGS is likely to improve in the future with the development of computational algorithms for the analysis of massive data and with the advancing comprehension of the function of non-coding DNA.

Reporting candidate variants in CHD studies is still far from delivering the results to patients in the clinics, as robust evidence for pathogenicity must exist to interpret the variant as pathogenic. The American College of Medical Genetics and Genomics (ACMG) has developed a detailed algorithm for the interpretation of sequence variants [45]. For novel genes without a validated association to a patient’s phenotype, mechanistic data would be needed before the gene can be classified as a disease gene. This can be done, for example, by establishing pathogenicity in model animal studies, assaying enzymatic function directly from biopsied tissue from the patient, or more recently using hiPSCs based disease models. This is often not possible in the clinical setting. However, while variant interpretation is cumbersome, the advantage in WES and WGS is that the data can be re-evaluated or re-analyzed later as new disease-associated variants are being reported.

In diagnostics, often subsets of genes, so-called gene panels, known to be relevant to the phenotype are analyzed. Commercial gene panels are also offered for CHD [46]. As most of the previously reported pathogenic variants in CHD have been private for individuals and families, the same difficulties prevail in variant interpretation as in WES and WGS. Of novel variants, only putative loss of function variants (nonsense, splicing, or frameshift variants) in genes where haploinsufficiency is not tolerated can be directly classified as pathogenic or likely pathogenic. Thus, it is likely that the analyses will result in a high number of variants of uncertain significance (VUS) causing confusion in families. While gene panels function well for other inherited cardiovascular diseases, such as LQTS or cardiomyopathies, where the same pathogenic variants recur, their benefit currently is slim in nsCHD.

## 4. Genome-Wide Association Studies

As genome-wide sequencing data is becoming available for an increasing number of populations, a commonly used method of studying the genetic background of traits or diseases, such as CHD is Genome-wide association studies (GWAS) [47,48,49]. Generally, GWASs compare the sequence data, or single nucleotide polymorphism (SNP) array intensities, of cases against controls to determine the effect sizes of genetic variants statistically, usually SNPs, on the trait in question. To date, there has been a multitude of GWAS studies on CHD [34,50,51,52,53,54,55]. However, the generally accepted threshold of genome-wide significance for GWASs (*p* < 5 × 10^−8^, [56]) has been difficult to achieve, often due to relatively small case-cohort sizes (usually less than 1000 individuals). This is further complicated by the relatively small effect size of common variants on phenotypes in general [57]. Thus, only a handful of studies have demonstrated genome-wide significant SNPs associated with CHD (Table 1). As it is known that different CHD lesions have at least partly distinct genetic etiologies, creating subtype categories of CHD can improve the analysis. For example, Lahm et al. [55] demonstrated a significant association of a large cohort of CHD patients with rs185531658, whereas in the same study, other SNPs showed significant association to various sub-groups of the cohort; transposition of the great arteries (TGA, six genome-wide significant variants), right heart lesions (one genome-wide significant variant), left heart lesions (one genome-wide significant variant), and anomalies of thoracic arteries and veins (ATAV, one genome-wide significant variant) [55]. Thus, it is important to define CHD subtypes considered during analysis clearly. The study by Lahm et al. [55] also combined GWAS data with hiPSC data and human and animal cellular level data to find support for the results. They showed that the gene with the strongest genome-wide significant SNPs, *MACROD2* (associated with TGA), was expressed during cardiac differentiation of hiPSCs, in mouse embryonic cardiogenic tissue and during human embryonic development within ventricular and outflow tract cells [55].

As additional GWASs are performed and the results are added to an already extensive body of literature, meta-analyses have become a standard approach to integrate data across different studies of the same trait [58,59]. By combining studies from different consortia, the sample size and statistical power may be increased. To date, there are multiple meta-analysis tools available, for example; METAL [59], PLINK [60], GWAMA [61], metafor [62], and meta [63]. Reports have indicated that meta-analysis provided by meta-analysis software is as efficient and powerful as pooling individual-level data across studies but more convenient and less bulky [59,63]. Furthermore, meta-analysis adds extra layers of analysis, enhancing the privacy of patients. Current meta-analysis methods also aim to address concerns regarding the differences in study designs, study cohorts (e.g., environment, linkage disequilibrium (LD), and ethnic composition), and chip/sequencing platforms between GWASs of the same trait [58].

## 5. Human Induced Pluripotent Stem Cells

**Figure 1:**
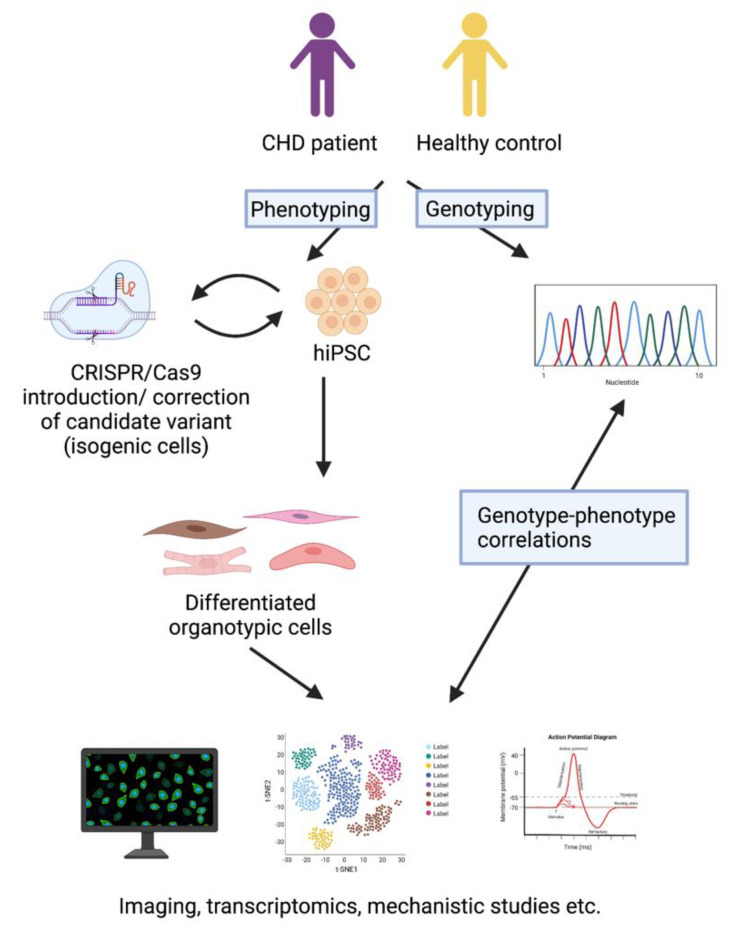
Disease modeling with human induced pluripotent stem cells (hiPSC). HiPSCs are derived from CHD patients and healthy controls. The candidate variant can be corrected with CRISPR-Cas9 technology in patient cells, or it can be introduced to cells from healthy controls. The hiPSCs can be differentiated to organotypic cells, such as cardiomyocytes, endothelial cells, fibroblasts, and smooth muscle cells. The phenotypes of the organotypic hiPSCs with the candidate variant can be compared to those without it, and genotype-phenotype associations can be identified. The figure has been created with BioRender.com.

The method of deriving hiPSCs from adult somatic cells [64], most commonly skin fibroblasts or peripheral blood mononuclear cells, provides the opportunity to study specific phenotypes in patient-derived cells [65]. Analyzing the phenotype and transcriptome of patient-derived hiPSCs that have been differentiated to organotypic cells can pinpoint specific genes or molecular pathways involved in the disease processes. This approach can be used for (1) identifying new candidate genes, (2) evaluating the pathogenicity of a new candidate disease variant, and (3) exploring the cellular and molecular consequences of a known disease-causing variant present in the patient (Figure 1).

HiPSC derived cardiomyocytes (hiPS-CM) have been widely used to model cardiomyopathies and rhythm disorders, and more recently, congenital heart defects. Hypoplastic left heart syndrome (HLHS) is the most commonly studied CHD phenotype [66,67,68,69], but also hiPSCs from patients with *NOTCH1*-associated bicuspid aortic valve (BAV) [70], *GATA4*-associated septal defects, and pulmonary stenosis ([71]), pulmonary atresia with intact ventricular septum [72], and elastin haploinsufficiency (Williams-Beuren syndrome) related supravalvular aortic stenosis and pulmonary stenosis [73,74] have been studied.

Most studies using HLHS patient-derived hiPSCs have focused on the cardiac myocyte phenotype in patients with both known or suspected (*NOTCH1*, *MYH6*) or unknown genetic etiology. Common findings from these studies have indicated that HLHS patient-derived cells have a diminished capacity for cardiomyocyte differentiation which has been documented on several stages during differentiation and a more immature hiPS-CM phenotype. The reduced expression of BRACHYURY and ISL1 during differentiation at day 7 suggests defects in the ability to generate mesodermal and cardiac progenitors, reduced expression of cTnT, SIRPA, HAND1, and HAND2 at day 14 suggests defects in generating cardiomyocytes, and the persistent expression of the cardiac progenitor marker GATA4 and reduced expression of cTnT at day 21 of differentiation indicates an immature cell type [67,75,76]. Other commonly reported findings include disorganized sarcomere structures [66,67,68,76,77], impaired Notch-signaling [66,67,68,76], and reduced expression of cardiac transcription factors, such as NKX2-5 and HAND1 [76] in hiPS-CMs derived from HLHS subjects. In addition, reduced nitric oxide signaling has been documented in HLHS hiPS-CMs [68].

All CHD disease phenotypes have not been replicated. For example, the lower differentiation efficiency of HLHS hiPS-CMs compared to controls is not noted in all studies [78]. Similarly, a lowered beating rate in HLHS hiPS-CMs [67,77] was not noted in one study [78]. On the other hand, some of the phenotypic properties shown in HLHS hiPS-CMs have been detected in hiPS-CMs of other CHD types. Impaired contraction in patient-derived hiPS-CMs has been reported in not only HLHS-derived hiPS-CMs [67,78] but also hiPS-CMs from family members with a pathogenic *GATA4*-variant causing septal defects and pulmonary stenosis [71], and hiPS-CMs from patients with pulmonary atresia with intact ventricular septum [72]. It is possible that distinct genetic etiologies contribute to the differences in phenotypes. It should, however, be kept in mind that phenotypic differences in the patient-derived hiPS-CMs might also reflect differences in experimental design and success, as there are many distinct differentiation protocols, and it is well known that large variations in differentiation efficiency occur [79]. Furthermore, the potential intra-clonal variation in hiPSC lines should be taken into account [80]. Moreover, it is known that hiPS-CM protocols can give rise to both atrial and ventricular-like cardiomyocytes, and protocols to direct the differentiation to each cell type have been established [79,81]. As these two cell types have different transcriptomic and electrophysiological properties, it is important to know and verify which cell type the used protocols produce. Single-cell RNA sequencing is a useful tool for investigating differentiation outcomes.

Besides hiPS-CMs, other cell types have also been studied. A recent study using HLHS-hiPSC-derived endocardial cells and hiPS-CMs indicated that developmentally impaired endocardium might result in abnormal endocardial to mesenchymal transition (EndoMT) and angiogenesis leading to ventricular and valvular hypoplasia in HLHS [68]. An impaired endocardial cell population was also detected in human heart tissue of fetuses with underdeveloped left ventricles. HiPS-derived smooth muscle cells (hiPS-SMCs) have been used to characterize molecular level mechanisms behind elastin deficiency causing supravalvular aortic stenosis and pulmonary stenosis. Elastin deficient hiPS-SMCs demonstrated immaturity with the lowered expression of differentiated SMC markers and less organized networks of smooth muscle α actin filament bundles [73,74] compared to hiPS-SMCs from healthy individuals. In addition, they showed increased proliferation and reduced response to vasoactive agonists [73,74], resembling the phenotype in vivo.

Knowing how variants affect the hiPS-CM phenotype can help in assessing the pathogenicity of variants of uncertain significance. A recent study demonstrated how the haploinsufficiency of *NAA15*, a known CHD gene, perturbs cellular function in hiPS-CMs. Heterozygous loss of function, compound heterozygous, and missense *NAA15* variants were introduced into hiPSCs using CRISPR/Cas9 gene editing, and the consequences of these mutations on hiPS-CM phenotype and RNA and protein expression were assessed [82]. The study identified four CHD-causing genes (*DHCR7*, *MAP2K2*, *NSD1*, and *RPL5*) that were differentially expressed in *NAA15* haploinsufficiency, providing molecular-level mechanisms behind the pathogenicity. Importantly, the study provided a reference on how to estimate the pathogenicity of *NAA15* variants of uncertain significance in patient-derived cells [82].

Recently more focus has been laid on the genetic determinants of disease course in CHD patients. For example, myocardial dysfunction is a known complication of HLHS where the right ventricle (RV) fails in an unexplained manner leading to the need for heart transplantation. Recent studies have indicated that variants in some cardiomyopathy genes, such as *MYH6*, *MYBPC3,* and *RYR2,* might predispose for such RV failure [34,83,84]. A recent study using hiPS-CMs from three HLHS subjects with early RV failure during the first decade of life demonstrated reduced contraction force and contraction acceleration compared with hiPS-CMs from healthy controls [78]. Furthermore, the hiPS-CMs had lower mitochondrial content and reduced oxygen consumption [78]. It was speculated that these functional impairments might contribute to susceptibility to early failure of the single RV in patients with HLHS. The study, however, did not analyze hIPS-CMs from HLHS subjects without RV failure. Thus, this could represent an overall phenotype of HLHS-hIPS-CMs. Still, this study demonstrates the potential of using hiPS-CMs in estimating disease course and prognosis.

When using hIPS-CMs for disease modeling, a relevant question is how the phenotypic changes in patients and hiPSC-CM disease models align with each other. HiPS-CM models for diseases, such as Long QT syndrome and hypertrophic and dilated cardiomyopathy, where the etiology of the pathologic process is in cardiomyocytes, recapitulate the electrophysiological, morphologic, and contractile phenotypes observed with the clinical phenotypes quite well [85]. Whether the hiPS-CM phenotypes resemble those of CHD patients’ cardiomyocytes in vivo is more difficult to assess. Generally, only little clinical information about the detailed phenotypes and cell model–patient phenotype correlations have been obtained from the CHD hiPS-CM studies. Heart development is a complex interplay between several different cell types and signaling pathways, where environmental factors, such as early blood flow and maternal metabolomics, also play a role [86]. Thus, it is apparent that a deficiency in cardiomyocytes is not solely, if at all, responsible for the observed complex phenotypes in CHD. The myocardial RV dysfunction observed in some HLHS patients can be due to several different causes, such as changes in hemodynamic stress during the different palliation stages, residual anatomic obstructions, arrhythmias, valve insufficiencies, myocardial ischemia, and genetic predisposition [87,88]. Thus, a thorough understanding of the developmental processes as well as of postnatal pathophysiological and hemodynamical conditions are essential when drawing conclusions on how cell models and patient phenotypes correlate in CHD.

Despite the limitations, using hiPSCs in disease modeling has several advantages compared to animal models. Animal models are more laborious, expensive, and may have ethical concerns. As patient-derived cells have the original genetic background, modeling is possible even with unknown or oligogenic etiology. Moreover, while animal models reflect the in vivo context better than cultured cells, the involved genes, developmental processes, and disease phenotypes are not always comparable between different species. Ongoing research on improving the differentiation efficiency and maturation of target cell types as well as developing cardiac organoids and 3D microtissues, will further enhance the use of patient-derived hiPSCs in disease modeling.

## 6. Conclusions

New sequencing technologies and large biobank sample collections have increased our understanding of the genetic basis of nsCHD. While some monogenic forms of CHD have been documented, the evidence supporting oligogenic inheritance in the majority of cases is accumulating. Curated lists of CHD-causing genes ranging from tens to thousands of genes [5,30] have been established, and even commercial gene panels are available [43]. However, since most of the thus far identified pathogenic variants seem to be specific to individuals or families, variant interpretation is not trivial. Further, as environmental exposures and epigenetic regulation contribute to CHD, the benefit of genetic diagnostics in nsCHD is not clear. Combining sample collections and data from biobanks to achieve larger patient cohorts and using innovative approaches, such as MR and advanced computation technologies, to evaluate the effects of common variants could clarify the inheritance patterns and reveal novel candidate loci. Information on genotype–phenotype associations from patient-derived organotypic hiPSCs provides an additional asset in evaluating candidate variants from WES and GWAS studies. Finally, the identification of gene variants affecting disease course provides additional impetus for research efforts, as these can guide disease follow-up and treatment and improve the prognosis of the CHD patient.

## Figures and Tables

**Table 1:** Collection of CHD GWAS studies, their significant loci, and nearest genes.

Study	Cohorts and CHD Subtype	Nearest Genes	Significant Loci
Cordell et al., 2013 [52] 835 cases, 5159 controls	Tetralogy of Fallot	*PTPN11*	1
Lin et al., 2015 [53] 945 cases, 1246 controls	Ventricular septal defect and/or atrial septal defect	*EDNRA*, *SMARCA2*, *TBX3*, *PTPRT*	4
Agopian et al., 2018 [54] 1940 cases and trios, 2976 controls	Conotruncal heart defect, left ventricular obstructive tract defect	*OFCC1*	1
Bjornsson et al., 2018 [56] 120 cases, 355,166 controls	Coarctation of the aorta	*MYH6*	1
Lahm et al., 2021 [55] 4034 cases, 8486 controls	All CHD phenotypes	KCNN2	1
	Transposition of the great arteries	*MACROD2*	6
	Right heart lesions	*SLC27A6*	1
	Left heart lesions	*ARHGEF4*	1
